# Comprehensive in-silico prediction of damage associated SNPs in Human Prolidase gene

**DOI:** 10.1038/s41598-018-27789-0

**Published:** 2018-06-21

**Authors:** Richa Bhatnager, Amita S. Dang

**Affiliations:** Centre For Medical Biotechnology, M. D. University, Rohtak, 124001 India

## Abstract

Prolidase is cytosolic manganese dependent exopeptidase responsible for the catabolism of imido di and tripeptides. Prolidase levels have been associated with a number of diseases such as bipolar disorder, erectile dysfunction and varied cancers. Single nucleotide polymorphism present in coding region of proteins (nsSNPs) has the potential to alter the primary structure as well as function of the protein. Hence, it becomes necessary to differentiate the potential harmful nsSNPs from the neutral ones. 19 nsSNPs were predicted as damaging by in-silico analysis of 298 nsSNPs retrieved from dbSNP database. Consurf analysis showed 18 out of 19 substitutions were present in the conserved regions. 4 substitutions (D276N, D287N, E412K, and G448R) that observed to have damaging effect are present in catalytic pocket. Four SNPs listed in splice site region were found to affect splicing of mRNA by altering acceptor site. On 3′UTR scan of 77 SNPs listed in SNP database, 9 SNPs were lead to alter miRNA target sites. These results provide a filtered data to explore the effect of uncharacterized nsSNP and SNP related to UTRs and splice site of prolidase to find their association with the disease susceptibility and to design the target dependent drugs for therapeutics.

## Introduction

Tissues are not only made up of cells, a valuable part of their volume is extracellular space, which is largely filled by a complex network of macromolecules constituting the extracellular matrix. This matrix is a well defined network of a variety of proteins and polysaccharides, which are in close association with the cell surface that secreted them. Collagen is the main component of extracellular matrix. Collagen is not only integral component of ECM but also has been known as a ligand for integrin receptors, playing an important role in signaling that regulate lipid metabolism, transport of ion, activation of various kinases and gene expression^1^. Therefore, any modification in the structure, quantity, and distribution of collagens in tissues affect a number of physiological processes like cell signaling, metabolism and function. Collagen catabolism involves the activity of various enzyme acting at different step. Its final step of degradation is the breakdown of imido dipeptides and tripeptides. Prolidase (E.C. 3.4.13.9) is a cytosolic exopeptidase that specifically cleaves imido dipeptides and imido tripeptides with C-terminal proline or hydroxyproline and releases free proline^2^. In this way prolidase recycles proline for collagen metabolism and serves as a rate limiting step in collagen metabolism. Any change in prolidase activity leads to disturbed collagen metabolism and results in diseased state^3,4^. Physiological levels of prolidase found to be associated with a number of diseases but still its exact role is obscure. It has been found that prolidase level is decreased to a significant extent in prolidase deficiency. Gene expression and post transcriptional modifications can have the potential to change the physiological level of prolidase. Prolidase gene (PEPD) is located on chromosome 19, contain 15 exon which encodes a polypeptide of 493 amino acids with molecular weight 54 kDa^5–7^. It is a dimer having two identical subunits. In humans, two isoforms of prolidase are present i.e. PDI and PDII. Nonsynonymous polymorphisms are those point mutations that insert amino acid change in the protein structure. Primary amino acid sequence is one of the factors which are responsible for mature protein structure as well as function of the protein. As these alterations can affect protein structure then it become important to study the effect of these polymorphisms on structure and function in detail and to figure out highly damaging mutation from the neutral one.

Most of the SNPs of prolidase are still uncharacterized in terms of their disease causing potential. From last few years, *in-silico* approaches have been widely employed to identify the impact of deleterious nsSNP in candidate genes by utilizing information like conservation of residues, structural attributes and physiochemical properties of peptides^8^. The *in silico* approaches offer advantages over the lab based characterization because of their reliability, convenience, speed, and of lower cost to find such variants that have the potential to regulate the function of prolidase protein^8,9^. So, present study has been carried out to extend and explore the effect of nsSNPs on the stability and function of the prolidase. Here we have used a set of computational techniques to prioritize the deleterious nsSNPs reported in the prolidase gene.

## Results

Prolidase is a well known dipeptidase which cleaves imino di and tripeptides containing proline. It possess both carboxypeptidase and aminopeptidase activity to cleave proline dipeptides. During final step of collagen degradation imido dipeptides are formed, prolidase degrades them and releases free proline for collagen resynthesis. Beside the dipeptidase function, prolidase also plays an important role as a detoxificant against chemical agents and pesticides. Human prolidase had a sequence similarity of 22% with OPAA (organophosphorus acid anhydrolase) which is also involved in the hydrolysis of pro-X combinations in Mn^2+^ dependent manner^10^. It also has been found that recombinant human prolidase also had both the activity i.e. hydrolysis of prolyl-glycyl peptides and digestion of organophosphate containing compounds^1^. Non synonymous mutation may leads to alteration in protein structure and function. In present analysis, sequence based, evolutionary and machine learning softwares were used to characterize the deleterious SNP from all the listed nsSNP in human.

### Retrieval of SNP ids

**Figure 1:**
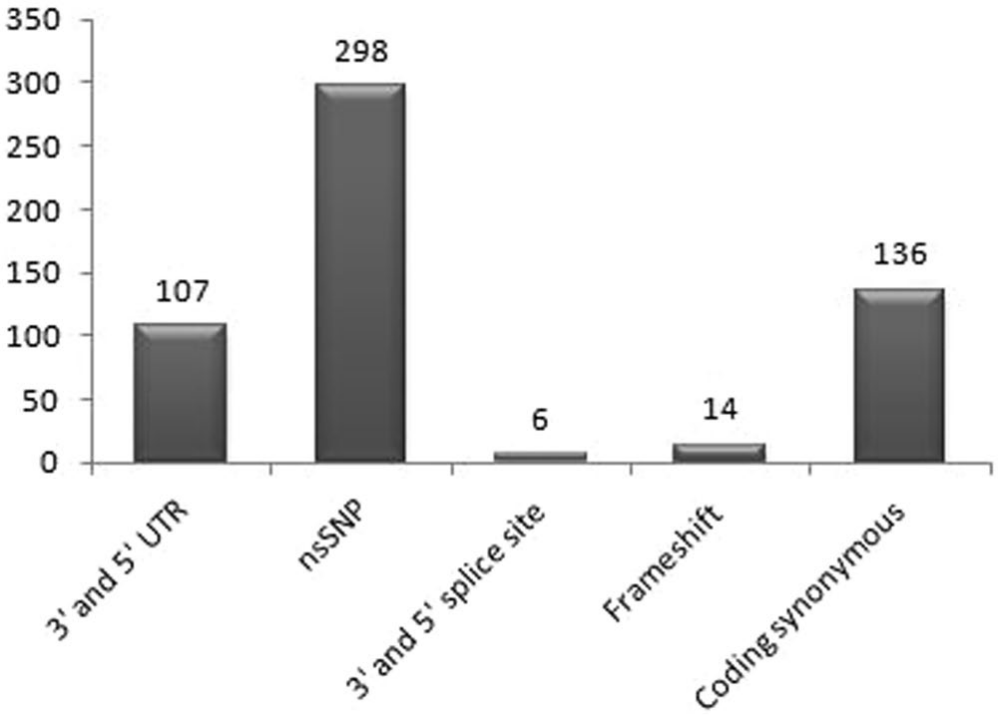
SNP distribution of Prolidase gene.

All the nsSNP were retrieved from dbSNP database (build150) using filters. A total of 26240 SNPs are reported in prolidase gene out of which 18308 SNPs are reported in Human prolidase gene. On further selection, 107 SNPs were found to be UTRs variants, 17730 as intron variants and 298 (292 missense, 6 nonsense) were nsSNPs (Fig. 1). By this data nsSNPs contribute to only 1.62% of all the SNPs reported in human prolidase gene. Protein ID used in analysis is NP_000276.1.

### Deleterious SNP prediction by SIFT

SIFT provides prediction for a list of nsSNP based on sequence homology and physical property of amino acids. It predicts whether the amino acid substitution at a given position is tolerated or not. This prediction is based on tolerance index (TI) where tolerance index is inversely proportional to the functional impact of substitution. rsids of 298 nsSNP were submitted for SIFT input and it predicted 39 substitutions as tolerated and 46 as deleterious as shown in (Table 1). Remaining 212 rsids were not found by SIFT server.

**Table 1:** Prediction of the effect of nsSNP by various tools.

S. No.	rsids	AAS	SIFT	FATHMM	Mutation Accessor	Provean	Polyphen	Phd-SNP	PANTHER	nsSNP	I-Mutant
1	rs17570	L435F	T	T	N	N	B	Dis	N	N	No effect
2	rs1063319	S247L	D	T	M	D	P.D	Dis	D	Dis	Decrease
3	rs1140312	D324V	D	T	N	N	B	N	D	N	Decrease
4	rs61734503	R33W	D	T	M	D	P.D	Dis	N	N	Decrease
5	rs61734505	R148C	T	T	L	D	B	Dis	N	N	Decrease
6	rs61734506	S103N	T	T	M	N	B	Dis	N	N	Decrease
7	rs61748998	E170V	T	T	N	D	B	—	N	N	Decrease
8	rs121917721	D276N	D	D	H	D	P.D	Dis	D	Dis	Decrease
9	rs121917722	R184Q	T	D	M	D	P.D	N	N	Dis	Decrease
10	rs121917723	G278D	D	D	H	D	P.D	Dis	D	Dis	Decrease
11	rs121917724	G448R	D	D	H	D	P.D	Dis	D	Dis	Decrease
12	rs139214756	S224T	D	T	M	D	P.D	N	N	N	Decrease
13	rs141623136	T188M	D	T	M	D	P.D	Dis	Dis	Dis	Decrease
14	rs142070498	D419G	D	T	M	D	B	N	N	N	Decrease
15	rs144944440	I418V	D	T	L	N	B	N	N	N	—
16	rs149042427	E391T	T	T	L	D	B	N	N	N	Decrease
17	rs151278946	D189G	T	T	M	D	—	Dis	N	N	Decrease
18	rs185183225	R35W	D	T	M	D	P.D	Dis	Dis	Dis	Decrease
19	rs186203899	T78S	T	T	L	N	B	N	N	N	Decrease
20	rs187269138	R33Q	T	T	L	N	B	N	N	N	Decrease
21	Rs188930796	T137M	T	T	L	N	B	Dis	—	N	Increase
22	rs189549581	V456M	D	T	M	N	P.D	N	N	N	Increase
23	rs199612179	C245C	T	T	M	N	—	Dis	N	N	—
24	rs199711203	A21V	T	T	L	D	B	N	N	N	Increase
25	rs199794147	E208V	D	T	L	D	B	Dis	N	N	Decrease
26	rs199892951	G51E	D	T	M	—	P.D	N	N	N	Increase
27	rs200072143	V472M	D	T	M	N	P.D	N	N	N	Decrease
28	rs200183031	D324L	D	T	—	N	B	N	N	N	Decrease
29	rs200351927	I201Q	T	T	M	D	P.D	Dis	N	Dis	Decrease
30	rs200435937	R35Q	T	T	L	N	B	Dis	N	N	Decrease
31	rs200450538	D189M	D	T	M	D	P.D	—	N	DIS	Decrease
32	rs200567073	G447R	D	D	M	D	P.D	Dis	N	Dis	Increase
33	rs200871513	G235S	T	T	H	D	P.D	N	D	Dis	Decrease
34	rs200931112	V305I	T	T	N	N	B	Dis	N	N	Decrease
35	rs201089253	G296E	D	D	M	D	P.D	Dis	D	Dis	Decrease
36	rs201222933	R398T	T	T	M	D	P.D	Dis	D	Dis	Increased
37	rs201447445	N250H	D	T	L	D	Ps.D	Dis	N	N	Increased
38	rs201572375	M210T	D	T	H	D	P.D	N	N	Dis	Decrease
39	rs201584435	D287N	D	D	H	D	P.D	Dis	D	Dis	Decrease
40	rs201752816	H72D	T	T	M	D	P.D	N	N	N	Decrease
41	rs201865747	D87N	T	T	M	D	B	Dis	N	N	Decrease
42	rs201992066	G12R	D	T	M	D	P.D	N	N	Dis	Decrease
43	rs267606943	S202F	D	T	H	D	P.D	N	N	Dis	Decrease
44	rs267606944	E412K	D	D	H	D	P.D	Dis	D	Dis	Decrease
45	rs367841505	D378N	D	D	H	D	P.D	Dis	D	Dis	Decrease
46	rs367902648	S240N	D	D	H	D	Ps.D	Dis	D	Dis	Decrease
47	rs368547324	G246S	T	T	L	D		Dis	N	Dis	Decrease
48	rs368559424	N151S	T	T	M	D	P.D	Dis	N	N	Decrease
49	rs368647287	R196C	T	T	H	D	P.D	N	N	Dis	Decrease
50	rs368651528	G381C	T	T	H	D	P.D	Dis	D	Dis	Increase
51	rs368784737	V171L	T	T	M	N	B	Dis	N	N	Decrease
52	rs368792538	N436N	T	T	L	N	N	Dis	N	N	Decrease
53	rs368995247	L66C	D	T	L	N	B	Dis	N	Dis	Decrease
54	rs369878645	I45V	T	T	N	N	B	N	N	N	Decrease
55	rs370219399	F275I	D	T	N	D	Ps.D	Dis	N	N	Decrease
56	rs370370158	K218T	D	T	N	D	B	N	N	N	Decrease
57	rs370970279	H255S	D	T	H	D	P.D	Dis	D	Dis	Decrease
58	rs371556469	A261R	T	T	L	D	P.D	Dis	N	N	Decrease
59	rs371934154	L403H	D	T	H	D	P.D	Dis	D	Dis	Decrease
60	rs371953949	L192W	D	T	M	D	P.D	Dis	Dis	Dis	Decrease
61	rs372210606	C290I	T	T	M	D	P.D	Dis	N	N	Decrease
62	rs372527759	R27Q	D	T	H	D	P.D	Dis	N	N	Decrease
63	rs372530277	G414S	T	T	—	D	P.D	N	D	Dis	Decrease
64	rs372629704	V181L	T	T	M	N	B	Dis	N	N	Decrease
65	rs373297406	G373H	T	D	H	D	P.D	Dis	D	Dis	Decrease
66	rs374162516	R196H	D	T	H	D	P.D	N	N	Dis	Decrease
67	rs374573875	F117L	T	T	M	D	B	N	N	N	Decrease
68	rs374603111	R335M	D	T	M	D	Ps.D	N	D	Dis	Increase
69	rs374795227	E227L	D	T	N	D	B	N	—	N	Increase
70	rs375061486	Y231C	D	T	M	D	Ps.D	N	N	Dis	Increase
71	rs375348295	S93L	D	T	M	D	P.D	Dis	N	N	Decrease
72	rs375915358	S142F	D	T	M	D	P.D	N	N	Dis	Increase
73	rs375919385	G323S	D	T	M	D	P.D	N	D	Dis	Decrease
74	rs376211407	I374K	T	T	M	D	Ps.D	Dis	D	Dis	Decrease
75	rs376338457	G260E	D	T	M	D	P.D	N	N	Dis	Decrease
76	rs376372688	G373C	D	T	H	D	P.D	N	D	Dis	Decrease
77	rs376397947	Y83C	T	T	M	D	Ps.D	N	N	N	Increase
78	rs376817734	R331C	T	T	M	N	B	Dis	N	N	Decrease
79	rs377085952	P19L	D	D	M	D	P.D	Dis	Dis	Dis	Increase
80	rs377199331	W326Y	D	T	L	—	P.D	N	N	N	Increase
81	rs377429945	D125N	T	T	L	N	B	Dis	—	N	Decrease
82	rs377536201	I329V	D	T	—	N	B	Dis	N	N	Increase
83	rs377685056	T410V	D	D	H	D	P.D	Dis	D	Dis	Increase
84	rs377714630	F169L	T	T	M	D	—	—	—	N	Decrease
85	rs377738544	S224I	D	T	M	D	B	Dis	Dis	Dis	Decrease

Abbreviations: T(tolerated), D(deleterious, damaging), N(neutral), Dis(disease causing), M(medium), L(low), H(high), B(benign), P.D(probably damaging), Ps.D(possibly damaging).

### Prediction of Functional effect of non synonymous SNP by Provean

Provean predicts the functional effect of amino acid substitutions. Threshold of prediction is −2.5, above this score prediction is supposed to be neutral and below or equal −2.5 prediction is deleterious. FASTA format with substitutions predicted by SIFT server were used as input. Out of 85 substitutions submitted, 21 amino acid substitution were predicted to be neutral (score is above-2.5) and remaining 64 were having score below or equal −2.5 and might be associated with disease (Table 1).

### Prediction of functional impact of mutation by mutation assessor

Mutation assessor calculates the impact of mutation on the function of protein. Its output results in FI score (functional impact combined score), VC score (variant conservation score), and VS score (variant specificity score). Functional impact categorized in two parts: predicted functional (having high and medium FI score) and predicted non functional (having low and neutral FI score). In this study, 19 mutations were found to be highly damaging, 38 having medium impact, 17 with low impact and 7 were classified as neutral (Table 1).

### Prediction of functional impact of nsSNP by PANTHER

PANTHER predicts the impact of mutation on the protein function. It uses HMM and various alignment method to map the mutation and then produces result. It gives result in the form of probability of damage associated with that SNP and noted as P_deletrious._ Minimum cutoff value is −3 for P_deletrious_ 0.5. Out of 85 nsSNPs, 21 were found to be damaging by PANTHER prediction (Table 1).

### Functional significance of substitution by Polyphen2

Polyphen i.e. polymorphism phenotype predicts the possible effect of amino acid substitution on function and structure of protein based on a number of criteria like phylogenetic, structural information and sequence of protein. It predicts sequence based feature on the basis of PSIC (position-specific independent count) matrix, TMHMM (transmembrane helix prediction by hidden markov model) algorithm, Coils2 program and SignalP program to predict transmembrane, coiled coil and signal peptide regions of the protein sequences and structure based feature on the basis of DSSP(dictionary of secondary structure protein) database. A (PSIC) score difference was assigned using the categories ‘probably damaging’, ‘possibly damaging’, and ‘potentially damaging’, ‘borderline’ and ‘benign’. Out of 85 nsSNP used in this study, 46 substitutions were probably damaging, 8 were possibly damaging and 31 were benign (neutral) in nature (Table 1).

### Disease associated SNP prediction by nsSNP analyzer and PhD SNP

Both nsSNP analyzer and PhD SNP predict the phenotypic effect of non synonymous substitution. They also predict whether the substitution is disease associated or not. By nsSNP prediction 40 substitutions are associated with disease whereas PhD SNP predicts 48 disease causing substitutions (Table 1).

### Prediction the effect of nsSNP by FATHMM

FATHMM depends upon hidden markov model about the pathogenicity of a substitution. It uses two different coordinates to make any prediction i.e. non coding variants and coding variants. Coding variants further differentiates into three part to be more specific in prediction i.e. inherited diseases (used to differentiates between disease causing mutation and neutral polymorphisms), cancer (used to differentiates between cancer promoting mutations and other germ line polymorphisms), disease specific (used to predict a list of potentially relevant SNPs for the disease of interest). FATHMM uses HMM and align the homologous sequences and conserved protein to give pathogenicity index about the mutation. In our analysis, 19 mutations were found to be damaging out of 85 mutations listed in the study (Table 1).

### Prediction of stability change by I-Mutant

The I-Mutant 2.0 server was developed and tested with the data extracted from ProTherm, to predict the change of protein stability due to mutation. Its prediction comes out in two forms i.e. change in DDG and ΔG. Positive G value leads to increased stability whereas negative G values correspond to decreased stability. Results of I- Mutant was summarized in Table 1.

### Consensus generation

To find the most deleterious SNP, concordance was done. Substitution which was predicted as deleterious by sequence and SVM based method were selected manually. A total of 19 substitutions were found to deleterious by all the algorithms used in the study as shown in Table 2.

**Table 2:** Consensus of all the softwares.

S No.	SNP	AAS	SIFT	Provean	Polyphen	nsSNP	PhD	I-Mutant	Consurf	NetsurfP
1.	rs377085952	P19L	DEL	DEL	Probably damaging	Disease	Disease	Increase	Conserved	Exposed
2.	rs185183225	R35W	DEL	DEL	Probably damaging	Disease	Disease	Decrease	Variable	Exposed
3.	rs141623136	T188M	DEL	DEL	Probably damaging	Disease	Disease	Decrease	Conserved	Exposed
4.	rs371953949	L192W	DEL	DEL	Probably damaging	Disease	Disease	Decrease	Intermediate	Buried
5.	rs377738544	S224I	DEL	DEL	benign	Disease	Disease	Decrease	Conserved	Exposed
6.	rs367902648	S240N	DEL	DEL	Possibly damaging	Disease	Disease	Decrease	Conserved	Buried
7.	rs1063319	S247L	DEL	DEL	Probably damaging	Disease	Disease	Decrease	Conserved	Buried
8.	rs370970279	H255S	DEL	DEL	Probably damaging	Disease	Disease	Decrease	Most conserved	Buried
9.	rs121917721	D276N	DEL	DEL	Probably damaging	Disease	Disease	Decrease	Most conserved	Buried
10.	rs121917723	G278D	DEL	DEL	Probably damaging	Disease	Disease	Decrease	Most conserved	Buried
11.	rs201584435	D287N	DEL	DEL	Probably damaging	Disease	Disease	Decrease	Most conserved	Buried
12.	rs201089253	G296E	DEL	DEL	Probably damaging	Disease	Disease	Decrease	Most conserved	Exposed
13.	rs373297406	G373H	DEL	DEL	Probably damaging	Disease	Disease	Decrease	Most conserved	Buried
14.	rs367841505	D378N	DEL	DEL	Probably damaging	Disease	Disease	Decrease	Most conserved	Buried
15.	rs371934154	L403H	DEL	DEL	Probably damaging	Disease	Disease	Decrease	Most conserved	Buried
16.	rs377685056	T410V	DEL	DEL	Probably damaging	Disease	Disease	Increase	Most conserved	Buried
17.	rs267606944	E412K	DEL	DEL	Probably damaging	Disease	Disease	Decrease	Most conserved	Buried
18.	rs200567073	G447R	DEL	DEL	Probably damaging	Disease	Disease	Increase	Most conserved	Buried
19.	rs121917724	G448R	DEL	DEL	Probably damaging	Disease	Disease	Decrease	Most conserved	Buried

### Prediction of association of substitution with disease by Mutpred

It predicts whether the nsSNP will be disease-causing or neutral^11^. It predicts the molecular cause of disease/deleterious. Its score is the probability that predict whether the substitution affects the function of protein or not. Threshold is 0.5: higher than 0.5 could be considered as ‘harmful’, whereas >0.75 could be considered a high confidence ‘harmful’ prediction. Prediction for the SNPs of prolidase is summarized in Table 3.

**Table 3:** Effect of nsSNP on the structure and function of protein predicted by Mutpred.

**S.No**	**rsids**	**Substitution**	**Effect**
1.	rs377085952	P19L	*Gain of helix (P* = *0.0022)* *Loss of loop (P* = *0.0031)* *Gain of MoRF binding (P* = *0.0759)* *Loss of methylation at K17 (P* = *0.0844)* *Gain of ubiquitination at K17 (P* = *0.107)*
2.	rs185183225	R35W	*Gain of catalytic residue at P38 (P* = *0.0394)* *Loss of disorder (P* = *0.0427)* *Loss of methylation at R35 (P* = *0.1122)* *Loss of MoRF binding (P* = *0.1173)* *Gain of helix (P* = *0.1736)*
3.	rs141623136	T188M	*Loss of methylation at K187 (P* = *0.0313)* *Loss of ubiquitination at K187 (P* = *0.1191)* *Loss of helix (P* = *0.1299)* *Gain of sheet (P* = *0.1945)* *Gain of MoRF binding (P* = *0.2083)*
4.	rs371953949	L192W	*Gain of MoRF binding (P* = *0.0284)* *Gain of methylation at K187 (P* = *0.0627)* *Loss of ubiquitination at K187 (P* = *0.1037)* *Loss of catalytic residue at L192 (P* = *0.1737)* *Loss of stability (P* = *0.2356)*
5.	rs377738544	S224I	*Loss of disorder (P* = *0.0628)* *Loss of catalytic residue at S224 (P* = *0.0702)* *Loss of phosphorylation at Y220 (P* = *0.0771)* *Loss of helix (P* = *0.2022)* *Loss of MoRF binding (P* = *0.3016)*
6.	rs367902648	S240N	*Loss of catalytic residue at S240 (P* = *0.0353)* *Loss of disorder (P* = *0.0834)* *Loss of phosphorylation at S240 (P* = *0.116)* *Gain of sheet (P* = *0.1451)* *Loss of stability (P* = *0.3235)*
7.	rs1063319	S247L	*Loss of glycosylation at S247 (P* = *0.0118)* *Loss of disorder (P* = *0.0567)* *Gain of sheet (P* = *0.0827)* *Loss of loop (P* = *0.2237)* *Gain of stability (P* = *0.2614)*
8.	rs370970279	H255S	*Gain of catalytic residue at H255 (P* = *0.0558)* *Gain of disorder (P* = *0.0697)* *Loss of sheet (P* = *0.302)* *Gain of glycosylation at S251 (P* = *0.315)* *Loss of stability (P* = *0.4182)*
9.	rs121917721	D276N	*Loss of sheet (P* = *0.0817)* *Loss of phosphorylation at Y281 (P* = *0.1679)* *Loss of stability (P* = *0.3001)* *Gain of catalytic residue at D271 (P* = *0.439)* *Loss of disorder (P* = *0.6276)*
10.	rs121917723	G278D	*Loss of catalytic residue at D276 (P* = *0.0909)* *Gain of sheet (P* = *0.1208)* *Gain of phosphorylation at Y281 (P* = *0.2344)* *Loss of stability (P* = *0.4985)* *Gain of disorder (P* = *0.6248)*
11.	rs201584435	D287N	*Gain of sheet (P* = *0.1208)* *Loss of loop (P* = *0.2237)* *Loss of catalytic residue at D287 (P* = *0.229)* *Loss of stability (P* = *0.2971)* *Gain of MoRF binding (P* = *0.3741)*
12.	rs201089253	G296E	*Gain of disorder (P* = *0.0902)* *Gain of sheet (P* = *0.1539)* *Gain of solvent accessibility (P* = *0.1683)* *Loss of catalytic residue at K297 (P* = *0.1817)* *Loss of helix (P* = *0.2022)*
13.	rs373297406	G373H	*Loss of sheet (P* = *0.1158)* *Loss of stability (P* = *0.2508)* *Gain of loop (P* = *0.2754)* *Gain of catalytic residue at G373 (P* = *0.3313)* *Gain of disorder (P* = *0.4695)*
14.	rs367841505	D378N	*Gain of sheet (P* = *0.0827)* *Loss of disorder (P* = *0.1773)* *Gain of loop (P* = *0.2754)* *Loss of phosphorylation at Y382 (P* = *0.3328)* *Loss of catalytic residue at G380 (P* = *0.5121)*
15.	rs371934154	L403H	*Gain of disorder (P* = *0.0202)* *Loss of stability (P* = *0.0827)* *Loss of sheet (P* = *0.1158)* *Gain of catalytic residue at R401 (P* = *0.1741)* *Gain of loop (P* = *0.2045)*
16.	rs377685056	T410V	*Loss of sheet (P* = *0.1907)* *Loss of catalytic residue at T410 (P* = *0.3448)* *Gain of loop (P* = *0.3485)* *Gain of MoRF binding (P* = *0.4771)* *Loss of glycosylation at T410 (P* = *0.5011)*
17.	rs267606944	E412K	*Gain of methylation at E412 (P* = *0.0028)* *Gain of ubiquitination at E412 (P* = *0.0408)* *Loss of sheet (P* = *0.0817)* *Gain of MoRF binding (P* = *0.1652)* *Gain of loop (P* = *0.2754)*
18.	rs200567073	G447R	*Gain of MoRF binding (P* = *0.0245)* *Gain of sheet (P* = *0.039)* *Gain of methylation at G447 (P* = *0.0399)* *Gain of loop (P* = *0.0435)* *Gain of solvent accessibility (P* = *0.0584)*
19.	rs121917724	G448R	*Gain of MoRF binding (P* = *0.0193)* *Gain of sheet (P* = *0.0827)* *Loss of catalytic residue at V449 (P* = *0.0969)* *Gain of methylation at R444 (P* = *0.1378)* *Loss of helix (P* = *0.2022)*

### Prediction of conserved and solvent accessibility by Consurf and NetSurf P

Consurf gives the output in the form of score where score 9 represent the most conserved and 1 represent the highly variable amino acid as given in Table 2. NetSurf P prediction about solvent accessibility (exposed, buried, and partially buried) for the amino acid substitution is also given in Table 2.

### Prediction of the effect of SNP located in UTR region by UTRscan Server and PolymiTRS

UTRscan server predicted the effect of UTRs on transcriptional motif. FASTA format of prolidase protein or UTRscan prediction and it predicted one signal in uoRF (Upstream Open Reading Frame) with a match 4 in 5′UTR region. PolymiRTS was employed to screen the effect of 3′UTRs on miRNA target site. It predicted 9 mutations have the potential to alter miRNA seed region. Out of these 9 mutations, 5 were INDELS whose ancestral allele cannot be determined yet but alter the miRNA target site and remaining 4 (rs140038783, rs3556, rs149914845, rs77690463) were SNPs which creates new target miRNA site as shown in Table 4.

**Table 4:** Predicted results of functional 3′UTR SNPs/Indels.

S.No	SNP ID	Allele	miR ID	miRSite	Function Class	context+score change
1	rs140842	ACTTT	hsa-miR-548av-5p	ctgaTACTTTActttctgtcaaaaat	O	−0.028
			hsa-miR-548k	ctgaTACTTTActttctgtcaaaaat	O	−0.028
			hsa-miR-548l	ctgATACTTTActttctgtcaaaaat	O	−0.083
			hsa-miR-8054	ctgaTACTTTActttctgtcaaaaat	O	−0.028
2	rs35012994	ACTTT	hsa-miR-548l	ttctgATACTTTctgtcaaaa	O	−0.041
3	rs71795604	TACTT	hsa-miR-548l	ttctgATACTTTctgtcaaaa	O	−0.041
4	rs10659604	TACTT	hsa-miR-548a-5p	gcatttctgaTACTTTActttctgtc	O	−0.097
			hsa-miR-548ab	gcatttctgaTACTTTActttctgtc	O	−0.097
			hsa-miR-548ak	gcatttctgaTACTTTActttctgtc	O	−0.076
			hsa-miR-548am-5p	gcatttctgaTACTTTActttctgtc	O	−0.085
			hsa-miR-548ap-5p	gcatttctgaTACTTTActttctgtc	O	−0.097
			hsa-miR-548aq-5p	gcatttctgaTACTTTActttctgtc	O	−0.088
			hsa-miR-548ar-5p	gcatttctgaTACTTTActttctgtc	O	−0.107
			hsa-miR-548as-5p	gcatttctgaTACTTTActttctgtc	O	−0.085
			hsa-miR-548au-5p	gcatttctgaTACTTTActttctgtc	O	−0.085
			hsa-miR-548av-5p	gcatttctgaTACTTTActttctgtc	O	−0.028
			hsa-miR-548ay-5p	gcatttctgaTACTTTActttctgtc	O	−0.085
			hsa-miR-548b-5p	gcatttctgaTACTTTActttctgtc	O	−0.076
			hsa-miR-548c-5p	gcatttctgaTACTTTActttctgtc	O	−0.085
			hsa-miR-548d-5p	gcatttctgaTACTTTActttctgtc	O	−0.085
			hsa-miR-548h-5p	gcatttctgaTACTTTActttctgtc	O	−0.076
			hsa-miR-548i	gcatttctgaTACTTTActttctgtc	O	−0.097
			hsa-miR-548j-5p	gcatttctgaTACTTTActttctgtc	O	−0.097
			hsa-miR-548k	gcatttctgaTACTTTActttctgtc	O	−0.028
			hsa-miR-548l	gcatttctgATACTTTActttctgtc	O	−0.083
			hsa-miR-548o-5p	gcatttctgaTACTTTActttctgtc	O	−0.085
			hsa-miR-548w	gcatttctgaTACTTTActttctgtc	O	−0.085
			hsa-miR-548y	gcatttctgaTACTTTActttctgtc	O	−0.088
			hsa-miR-559	gcatttctgaTACTTTActttctgtc	O	−0.1
			hsa-miR-8054	gcatttctgaTACTTTActttctgtc	O	−0.028
5	rs201816618	TCTGA	hsa-miR-548l	agcatttctgATACTTTctgt	O	−0.041
			hsa-miR-548a-5p	agcatTTACTTTctgt	O	−0.084
			hsa-miR-548ab	agcatTTACTTTctgt	O	−0.084
			hsa-miR-548ak	agcatTTACTTTctgt	O	−0.094
			hsa-miR-548am-5p	agcatTTACTTTctgt	O	−0.094
			hsa-miR-548ap-5p	agcatTTACTTTctgt	O	−0.112
			hsa-miR-548aq-5p	agcatTTACTTTctgt	O	−0.094
			hsa-miR-548ar-5p	agcatTTACTTTctgt	O	−0.094
			hsa-miR-548as-5p	agcatTTACTTTctgt	O	−0.084
			hsa-miR-548au-5p	agcatTTACTTTctgt	O	−0.094
			hsa-miR-548ay-5p	agcatTTACTTTctgt	O	−0.094
			hsa-miR-548b-5p	agcatTTACTTTctgt	O	−0.094
			hsa-miR-548c-5p	agcatTTACTTTctgt	O	−0.094
			hsa-miR-548d-5p	agcatTTACTTTctgt	O	−0.094
			hsa-miR-548h-5p	agcatTTACTTTctgt	O	−0.094
			hsa-miR-548i	agcatTTACTTTctgt	O	−0.084
			hsa-miR-548j-5p	agcatTTACTTTctgt	O	−0.112
			hsa-miR-548o-5p	agcatTTACTTTctgt	O	−0.094
			hsa-miR-548w	agcatTTACTTTctgt	O	−0.094
			hsa-miR-548y	agcatTTACTTTctgt	O	−0.094
			hsa-miR-559	agcatTTACTTTctgt	O	−0.103
6	rs140038783	A	hsa-miR-4310	gaaaatAATGCTG	D	−0.237
			hsa-miR-7157-5p	gaaaatAATGCTG	D	−0.237
		G	hsa-miR-1250-3p	GAAAATGAtgctg	C	−0.201
			hsa-miR-153-5p	gAAAATGAtgctg	C	0.023
7	rs3556	T	hsa-miR-3163	ctgTTTTATAcct	D	0.091
		C	hsa-miR-494-3p	cTGTTTCAtacct	C	−0.065
8	rs149914845	T	hsa-miR-105-5p	cgGCATTTGAtca	D	−0.167
			hsa-miR-7853-5p	cgGCATTTGAtca	D	−0.188
		C	hsa-miR-1245b-3p	cggCATCTGAtca	C	−0.074
			hsa-miR-383-5p	cggcaTCTGATCA	C	−0.29
			hsa-miR-4772-5p	cggcatCTGATCA	C	−0.091
9	rs77690463	C				
		T	hsa-miR-4719	tcttTTTGTGAtg	C	−0.002

miRSite: sequence context of the miRNA site: bases complementary to the seed region are in capital letters and SNPs are highlighted in bold font. Function class: D: the derived allele disrupts a conserved miRNA site (ancestral allele with support >2); C: the derived allele creates a new miRNA site; O: the ancestral allele cannot be determined. Context score: negative increase = increase in SNP functionality.

### Prediction the effect of SNP located in splice site by HSF tool

HSF tool analyse the effect of any mutation on splicing signals and recognize the splicing motifs in any human gene sequence. cDNA sequence containing point mutation or insertion or deletion was submitted to HSF server and it predicted 5 SNPs from 3′ and 5′ splicing region would alter the splicing signal. Out of these 5 mutations, 4 (rs542228812, rs753775083, rs761217488 and rs907881705) were found to affect splicing of mRNA by altering acceptor site whereas rs1016478683 affect splicing by affecting donor site (Table 5).

**Table 5:** Effect of 5′ and 3′ splice sites.

S.No	rsids	Predicted signal	Interpretation	Exon location
1.	rs542228812	Broken WT Acceptor Site	Alteration of the WT acceptor site, affecting splicing	7
2.	rs753775083	Broken WT Acceptor Site	Alteration of the WT acceptor site, most probably affecting splicing	7
3.	rs761217488	Broken WT Acceptor Site	Alteration of the WT acceptor site, most probably affecting splicing	11
4.	rs907881705	Broken WT Acceptor Site	Alteration of the WT acceptor site, most probably affecting splicing	6
5.	rs1016478683	Broken WT Donor Site	Alteration of the WT donor site, most probably affecting splicing.	14
6.	rs1055732229	—	Not found in HSF database	

### Secondary structure prediction by PSIPRED

Secondary structure of prolidase was predicted by PSIPRED which showed the distribution of alpha helix, beta sheet and coils. By analysis it was found that in native structure coils contribute major portion in protein structure (48.9%) followed by alpha helix (26.5%) and β- strand (24.4%) (see Supplementary File S1). On insertion of all the 4 (D276N, D287N, E412K, G448R) damaging substitutions, major distortion was loss of strand at residues 415 and 416 ((see Supplementary File S2).

### Three dimensional structure prediction by Swiss-Modeler

**Figure 2:**
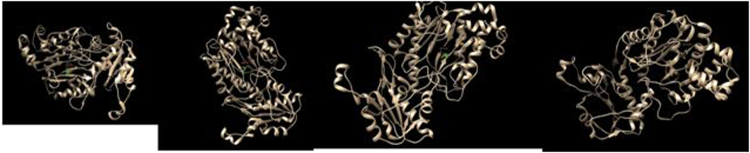
3D structure of native prolidase generated by Modeller and visualized by Pdb viewer.

**Figure 3:**
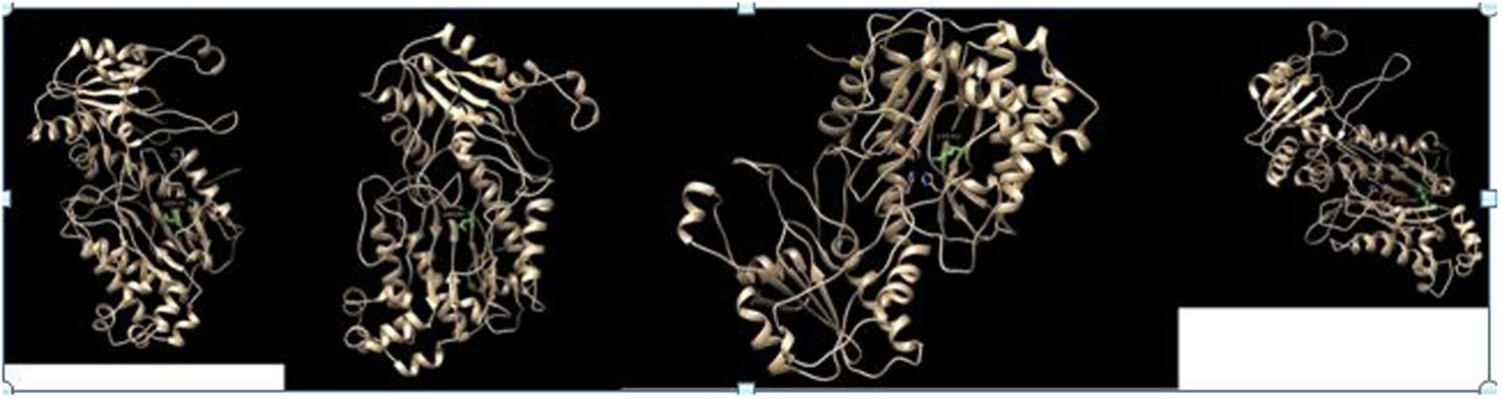
3D structure of D276N, D287N, E412K, G448R substituted prolidase generated by Modeller and visualized by Pdb viewer.

4 (D276N, D287N, E412K, G448R) models were generated by Swiss modeler for prolidase protein. Models with the Z-score between the ranges of 0–1 are considered as good models. Both the native and mutated models were further visualized and analyzed by UCSF Chimera (Figs 2 and 3). 3D structure of prolidase protein was of 493 amino acid residues. QMEAN, GMQE, RMSD values, energy minimization values and gradiant norms of mutated models are given in Table 6.

**Table 6:** Quality parameters of D276N, D287N, E412K, G448R substituted models.

	GMQE	QMEAN	RMSD (metre)	Potential Energy after minimization (Joules)	Gradient norm	RAMPAGE		
						Number of favorable region residues	Number of allowed region residues	outlier
D276N	0.99	−0.10	0.096019	−4254.726910	96.507714	467 (96.9%)	15 (3.1%)	0
D287N	0.99	0.32	0.082341	−42762.470954	88.725110	465 (97.1%)	13 (2.7%)	1 (0.2%)
E412K	0.99	0.10	0.100311	−44425.266536	88.393204	467 (96.9%)	15 (3.1%)	0
G448R	0.99	0.10	0.101967	−44357.73369	89.701027	468 (97.3)	13 (2.7%)	0

### Model validation by RAMPAGE

Quality of all the 4 (D276N, D287N, E412K, G448R) models was checked by RAMPAGE which is a indicative of Ramachandran plot. All the substituted models are of good quality as having more than 90% region in favoured region (Table 6). Quality assessment structure of RAMPAGE prediction are given as Supplementary File S3.

## Discussion

Prolidase, also known as Peptidase D or Iminopeptidase has been found in almost all the organism ranging from prokaryotes to eukaryotes^12–14^. The human enzyme is homodimeric, and found in two different isoforms i.e. PD I (higher activity against Gly-Pro dipeptides, depends on Mn^+2^ ion for catalysis) and PD II (higher activity against Met-Pro dipeptides and a little activity against Gly-Pro, requires Zn^+2^ for catalysis). In humans, PDI isoform is abundant and responsible for prolidase deficiency and collagen related disorders^14^.

This dimer has a crystal structure that shows two approximately symmetrical monomers, both have an N-terminal domain, made up of a six-stranded mixed β-sheet flanked by five α-helices, a helical linker, and C-terminal domain, consisting of a mixed six-stranded β-sheet flanked by four α-helices^15^.

Human prolidase protein has two domain i.e. domain ranging from 18–191 is aminopeptidase domain and 192–479 is M24 like hydrolases domain respectively. Its main activity i.e. proline dipeptidases activity is confined to a cluster around metal binding site with a conserved stretch ranging from 366–378^15^. Binuclear active metal site cluster which possess substrate binding site activates the nucleophiles and stabilize the transition state to facilitate transitions. Both the active site and metal cluster lies on the inner surface of the β-sheet of M24 domain which is anchored by the side chains of two aspartate residues (Asp276 and Asp287), two glutamate residues (Glu412 and Glu452), and a histidine residue (His370). Carboxylate group of aspartate and glutamine residues serve as bridges between the two Mn atoms as shown by PDB.

Function of protein directly depends on its tertiary structure thereby modification in the amino acid may have potential to alter protein structure and can produce severe physiological effects. Alteration in physiological level of prolidase affects the final step of collagen metabolism and can cause collagen related disorders. A well known pathological condition, Prolidase deficiency is characterized by skin ulcers, micrognathia, and hypertelorism. Increased physiological levels of prolidase have been found in cardiac diseases, bipolar disorder, depression, erectile disorder, and in a number of cancer whereas in asthma, COPD, osteoarthritis, chronic pancreatitis, and in pancreatic cancer its levels were found to be decreased^11,16–24^.

*In-silico* analysis provides us a key to predict the effect of single nucleotide polymorphism on the structure and function of a protein^25^. We used algorithms based on sequence and structure along with machine learning methods to deduce the effect of nsSNP on prolidase structure and function.

298 SNPs retrieved from dbSNP were submitted for SIFT prediction to deduce the amino acid substitution caused by these SNPs. SIFT predicted 85 substitutions that caused amino acid change based on the degree of conservation of amino acid residues in sequence alignments derived from closely related sequences, collected through PSI-BLAST. SIFT predicted 46 out of these 85 substitutions were deleterious in nature while other were neutral. These 85 substitutions were analyzed further to conclude their effect on protein structure and function.

Provean predicted 64 substitutions to be damaging. Structural impact of non synonymous mutations was predicted by Polyphen2 program which predicted 46 substitutions were probably damaging, 8 possibly damaging and remaining substitutions not having any impact on protein structure. Mutation accessor, predicted 57substitutions to be damaging. nsSNP and PhD server were also employed to check the effect of these substitutions and they predicted 40 and 48 substitutions damaging respectively.

Manual concurrence of all the SNPs studied by different softwares was done. Total 19 substitutions were found common in all the softwares used in the study. Effect of these nonsynonymous mutations on stability was checked by I-Mutant server which gives the prediction in the form of DDG. I- mutant predicted 16 out of 19 substitutions decrease the stability of protein whereas 3 substitutions (G447R, P19L, T410V) were found to make protein more stable

Consurf predicted that out of these 19 substituted positions, 18 are highly conserved in prolidase structure (Table 2). P19, R35, T188, G296 and G447 positions are exposed in the prolidase structure while remaining 14 are buried inside as predicted by NetSurfP. These substitutions can be segregated on the basis of domain where they are found. 3 substitutions are present in aminopeptidase domain while 16 are located in M24 like domain. Furthermore out of 16 substitutions in M24 domain, 3 substitutions (D276N, D278N, E412K) are present in metal binding site.

P19L entails a substitution of proline by leucine. This substitution leads to increased aggregation tendency but decrease the chaperone binding affinity. It also leads to alteration in structure by increasing the tendency to form a helix. It also generate site for ubiquitinylation making the region prone to degradation and decrease the stability thereby affecting the physiological level of prolidase.

R35W mark the substitution of arginine (basic amino acid) by tryptophan (a non polar aromatic amino acid). This residue involves in the formation of helix and interacts with P38. By loss of arginine, methylation and MoRF binding activity was found to be lost as predicted by Mutpred. It also leads to gain of catalytic activity at P38 residue but decrease the stability of protein. T188M involves the substitution of theronine (polar) to methionine (non polar) although increase the stability of protein by loss of ubiquitinylation site to make protein more stable but results in loss of methylation and helix formation property. Proline dipeptidase activity of prolidase is dependent on the phosphorylation of serine/Threonine residues. Methylated serine/Threonine residues might serve as the recognition site for serine/theronine kinase resulting in pro-dipeptidase activity. Loss of methylation at ‘T’ donot confers the recognition site for kinase and decreases prolidase activity. This substitution also leads to formation of β- strand thereby altering the protein structure. Both P19L and R35W if present would to lead to disruption of aminopeptidase domain and T188 leads to decreased activity.

In M24 domain, 2 SNPs leads to substitutions of leucine (L192W, L403H) by tryptophan and histidine respectively where former belong to non polar group and histidine belong to basic charged amino acid. In L192W both amino acids are non polar in nature but this substitution leads to disruption of helix because of bulky nature of tryptophan which don’t fit inside the helix. Both of these substitutions also leads to loss of chaperone binding affinity, decrease in stability of helix resulting loss of catalytic site.

3 substitutions are related to replacement of serine (S224I, S240N, S247L). They involves the substitution of serine (-OH containing amino acid) to isoleucine (non polar amino acid), arginine (Basic amino acid) and leucine respectively. All three regions forms strand in protein structure. S224I substitution increases the protein stability but results in loss of catalytic residue S at this region. Besides this, this substitution also influences the phosphorylation of tyrosine residue at 220^th^ position leading to the loss of activity of this domain. S240N and S247L both decrease the stability of protein, loss of catalytic property thereby making the protein non functional. S247L substitution also leads to loss of glycosylation at 247^th^ position resulting in altered catalytic site of protein.H255S substitution leads to decrease in the protein stability. It involves the substitution of Histidine (basic amino acid) to serine (OH containing amino acid) this substitution disrupt the secondary structure of protein.

As deduce by the study of Roberta Besio *et al*.^26^, it was found that Asp 276, Asp 287, His370 and Glu 412, 452 forms the catalytic site responsible for di-peptidase activity of the prolidase. Asp 287 and Glu 452 forms the binding site for Mn_1_ and Mn_2_ ion in subunit A and B as well. Glu412 binds with Mn_1_ and Asp 276 binds with Mn_2_ in both the subunits whereas His370 binds only Mn_1_ in subunit B. Theronine residues were found to be more conserved near this catalytic site. T289 residue helps in binding with Mn_2_ whereas T410 found in the site bind with Mn_1_^26^. Any mutation in this region would lead to loss of di-peptidase activity and contribute to prolidase deficiency. Our results also suggest that substitutions in these residues may have damaging effects. D276N decrease the protein stability, loss in strand formation and phosphorylation at Y281 residue. G278D results in loss of catalytic activity of the residue D276 but gain of phosphorylation at Y281 as predicted by Mutpred. This alteration makes the catalytic site nonfunctional. G296E and G373H substitution severely reduces the stability of protein. This substitution increases the solvent accessibility making the buried region to expose and destabilizing the structure with the loss of catalytic residue at K297. Mn(II) ions in the catalytic site are surrounded by negatively charged amino acids aspartic acid and Glutamic acid (D276, D287, E412, E452) and a phosphate group. E412K mutation decreases the negative charge by two units in the coordination sphere making it non functional. Furthermore, E412K substitution increases the aggregation tendency of protein but decrease chaperone binding property responsible for proper folding of the protein. This substitution makes the protein prone to ubiquitinylation and results in loss of strand from the protein structure. G447R and G448R substitutions both results in loss of prolidase activity. Residue G448 is inaccessible to solvent because it is buried inside the protein region. The residue lies at about 14.5 A° from the active site and is not directly involved in Mn(II) binding. G448 is a part of anti-parallel β strand combined with a short strand composed made up of residues G414, I415, Y416, F417. The G448R substitution leads the insertion of a bulky arginine side chain which is not appropriate with pairing of the two anti-parallel β strands and with the correct assembly of the b-sheet. Furthermore, the G448R mutation falls only four amino acids before residue E452, that coordinates one of the Mn(II) cofactor ions; thereby disrupting the catalytic site for di- peptidase activity. Residues ranging from 366–378 are highly conserved and results in proline di- peptidase activity. All the above listed substitutions lead to decrease in prolidase activity either by disrupting its structure or by loss of proper catalysis and phosphorylation at the sites needed for its activity.

Secondary structure of native prolidase and mutation incorporated (D276N, D287N, E412K, G448R) prolidase reveals no such considerable variation. But these substitutions affect the tertiary structure of protein as being a part of catalytic site. Therefore it can predict that these 4 substitutions have potential to affect the function of prolidase protein.

## Conclusion

Prolidase is an important regulator of collagen metabolism. A number of studies are present on prolidase deficiency, a rare autosomal recessive disorder. But there is lack of studies related to prolidase on molecular level. Almost all of the SNPs are still uncharacterized in their disease causing potential except those for related to prolidase deficiency. This is the first study which predicts the functional and structural impact of nsSNP on prolidase structure and function. This study differentiates disease causing mutations from neutral ones as listed in SNP database. Furthermore, the predicted disease associated nsSNP can be studied to find their association in various disease development and development in potent drug discovery. In addition to this, results of present study should be updated in relevant database so that other can use these results to make further studies^27–30^.

## Materials and Methods

### SNP retrieval

SNP of prolidase gene and their protein sequence (FASTA format) were retrieved from dbSNP database (http://www.ncbi.nlm.nih.gov/SNP/) and NCBI respectively for computational analysis. Selection of SNPs related to *Homo sapiens* was done by using filters non synonymous, missense, nonsense, stop gained SNP and human^31^. Other databases such as Exome Aggregation Consortium (ExAC), Genome Variation Server (GVS) and F-SNP were also searched to cross check the nsSNP data for prolidase gene.

### Prediction of the effect of nsSNPs

nsSNPs carried out amino acid substitution was first screened by SIFT(Sorting Intolerant from Tolerant) server. Its prediction is based on the conservation and alignment of highly similar orthologoue and paralogoue protein sequences and predict the functional importance of an amino acid substitution. Positions with probability score less than 0.05 are considered to be deleterious, those greater than or equal to 0.05 are considered to be tolerated^32^. In our study, we submitted rsids retrieved from dbSNP as a query to make prediction. nsSNPs prediction by SIFT server was further used to find their effect on the structure and fnction of prolidase gene. Protein variation effect analyzer(PROVEAN) predicts whether the substitution of amino acid is deleterious or tolerated. The threshold for a mutation to be deleterious is −2.5; if below threshold, prediction will be deleterious and will be neutral if it is above threshold. Provean program can be used to predict a functional effect of single or multiple amino acid substitutions, insertions or deletion^10^.

Mutation Assessor predicts the effect amino‐acid substitutions on the function of proteins by utilizing a combinatorial entropy optimization’ technique to find key residues responsible for function and then assigns a conservation score to them. This server provides semantic linking to variant analysis, annotations, variant multiple sequence alignment html page, and variant 3D structure page. Its output contains two annotation i.e. FI score (functional impact score) and functional impact (high, medium, neutral). PANTHER is a mutation analysis software that depends upon the HMM to make any prediction. It has three variants: gene list analysis, panther scoring, and evolutionary analysis of coding SNPs. In gene list analysis, it analyzes the list of gene, and expression data files with PANTHER. By Evolutionary analysis of coding SNPs it predicted the chances of a particular nonsynonymous coding SNP will cause a functional impact on the protein or not. Polyphen2 predict the functional impact of single amino acid substitution on protein function using physical and comparative models generate by the sequence information. Its prediction is based on a number of features such as sequence, structure and phylogenetic comparison to analyze the mutation^33^. PhDSNP is support vector machine based software which support the local sequence environment and output of multiple sequence alignment to predict the nature of a particular mutation. It requires input in the form of protein sequence, residue position, new residue^34^. Output is based on reliability score which predict whether the substitution is disease causing or neutral. nsSNP analyser predicts the phenotypic effect of nonsynonymous substitution. It uses multiple sequence alignment and protein 3D structure to predict the result. nsSNP Analyzer uses “Random Forest” network i.e. a machine learning method to classify the nsSNP from native one. Its prediction is purely dependent on swissprot database and was trained using a curated SNP dataset. nsSNP Analyzer summarizes the structural environment of the mutated residue and similarity between the substituted and native residue from the normalized probability of the substitution in the multiple sequence alignment^35^. FATHMM uses hidden Markov models (HMMs) to predict the functional effects of protein missense mutations and assign a pathogenicity score representing the overall tolerance of the protein/domain to mutations. A consensus of all the predictions was generated to prioritize the deleterious substitution predicted by various softwares used. It was done by manual method. Results of all the software were analyzed and substitution were selected which are found to be deleterious in all the predictions. All the prioritized nsSNP were further studied by MutPred server which is a web tool that predicted nsSNP association with disease along with molecular effect of that particular substitution. It takes the input as SIFT output and calculate 14 different structural and functional properties. It was trained utilizing the deleterious mutations reported in Human Gene Mutation Database and neutral polymorphisms from Swiss-Prot. It uses SIFT, PSI-BLAST, and Pfam profiles^36^, also some structural disorder prediction algorithms, including TMHMM, MARCOIL^37^, and DisProt^38^. It uses SVM v2.50 for analysis The output of MutPred consists of a general score (g), i.e., P (deleterious) the probability that the amino acid substitution is deleterious or disease-associated, and top five characteristic scores (p), where p is the P-value that certain functional and structural characteristics of the protein are impacted. Certain combinations of high values of ‘g’ (p deleterious) and low values of ‘p’ (property scores) are referred as hypotheses. • Scores for an aas with g > 0.5 and p < 0.05, are referred as actionable hypotheses. • Scores for an aas with g > 0.75 and p < 0.05, are referred as confident hypotheses. • Scores for an aas with g > 0.75 and p < 0.01, are referred as very confident hypotheses. User input involves FASTA sequence and amino acid substitutions.

### Prediction of conserved residues by ConSurf

It calculates the evolutionary conservation of amino acid within a protein sequence by using empirical Bayesian inference. It gives conservation score along with color scheme. Score 9 was given to most conserved amino acid whereas 1 is given to variable amino acid^39^, Consurf is available at http://consurf.tau.ac.il/.

### Prediction on surface and solvent accessibility by NetSurf P

It predicts the solvent accessible surface area or solvent accessibility of amino acids to locate the active site in a fully folded protein. This prediction method relies on the *Z*-score, which can predict the surfaces but not secondary structures of proteins. Its ouput includes 3 subclasses meant for buried, partial buried and exposed region in protei structure^40^, www.cbs.dtu.dk/services/NetSurfP/.

### Prediction of stability change by I-Mutant

A support vector machine based tool iMutant 2.0 predicts the change in the stability of the protein by a particular mutation. iMutant 2.0 can be utilized both as a classifier that predicts the signs of the protein stability changes upon a variation and as a regression estimator that predicts the relative change in Gibbs-free energy (ΔG) at a given temperature. It utilizes a comprehensive database based on protein mutation ProTherm^41^, http://folding.biofold.org/i-mutant/i-mutant2.0.html.

### Prediction of the effect of SNP located in UTR region by UTRscan Server

Untranslated regions have considerable role in the post transcriptional regulation of gene expression, stability and efficiency of translation. UTRscan server predicts the functional SNPs by BLAST search to find UTR motifs present in UTRsite^42^. Its input format requires submission of protein’s FASTA format and output was in the form of signal name and its position in the transcript, http://itbtools.ba.itb.cnr.it/utrscan.

### Functionally significant 3′UTR prediction by PolymiRTS

Polymorphism in microRNA Target Site (PolymiRTS) is a repository of naturally occurring DNA mutations in the miRNA target site^43^. It predicts whether a point mutation or INDELS in 3′UTR affect the miRNA target site or not. Output was in the form of 4 categories i.e. ‘D’ (the derived allele disrupts a conserved miRNA site), ‘N’ (the derived allele disrupts a nonconserved miRNA site), ‘C’ (the derived allele creates a new miRNA site) and ‘O’ (other cases when the ancestral allele cannot be determined unambiguously) where class ‘C’ may cause abnormal gene repression and class ‘D’ may cause loss of normal repression control. These two classes of PolymiRTS are most likely to have functional impacts, http://compbio.uthsc.edu/miRSNP/.

### Prediction the effect of SNP located in splice site by HSF tool

Human splicing finder(HSF) identify and predicts the effect of mutations on the splicing motifs including the acceptor and donor splice sites, the branch point and auxiliary sequences known to either enhance or repress splicing: Exonic Splicing Enhancers (ESE) and Exonic Splicing Silencers (ESS)^44^, http://www.umd.be/HSF3/HSF.shtml.

### Secondary structure prediction by PSIPRED

PSIPRED (PSI BLAST based secondary structure prediction) predicted secondary structure of protein based on related sequences and position specific scoring matrix. It predicted whether the residues were form strand, helix and coils. Input format was the FASTA sequence of prolidase protein, http://bioinf.cs.ucl.ac.uk/psipred/.

### Three dimensional structure prediction by Swiss Model

Prediction of 3D structure was done by Swiss Modeller which allow to model the amino acid on the basis of structure homology. It allows modeling using manual template selection or by automated selection mode. It identifies the template, align the sequence, generate model then assess the model quality in terms of QMEAN value. FASTA sequence (mutation incorporated) was modeled against PDB structure of prolidase rprotein. Swiss Pdb viewer, tool was used to visualize and energy minimization of generated model, https://swissmodel.expasy.org/.

### Quality assessment by RAMPAGE

RAMPAGE is a web server predicted dihedral angles and number of residues in allowed, favorable region based on the Φ and Ψ angles. Pdb files of models obtained after energy minimization was used as input of RAMPAGE online tool. More than 90% residues in allowed region is considered as good model.

## Electronic supplementary material

Supplementary information
