# Supplementary material for: Comprehensive in-silico prediction of damage associated SNPs in Human Prolidase gene
Source: Sci Rep. 2018 Jun 21;8:9430. doi: 10.1038/s41598-018-27789-0 (PMC6013436; doi:10.1038/s41598-018-27789-0)
Supplement: Supplementary file 1 — Supplementary information [file 41598_2018_27789_MOESM1_ESM.pdf]

Title page

Title of manuscript: Comprehensive in-silico prediction of damage associated SNPs in Human Prolidase gene

Author list: Richa Bhatnager and Amita S. Dang*

Supplementary information:

S1: Secondary structure of prolidase protein

S2: Secondary structure of prolidase protein after incorporation of D276N, D287N, E412K, G448R substitutions

S3: RAMPAGE images of D276N, D287N, E412K, G448R substituted models

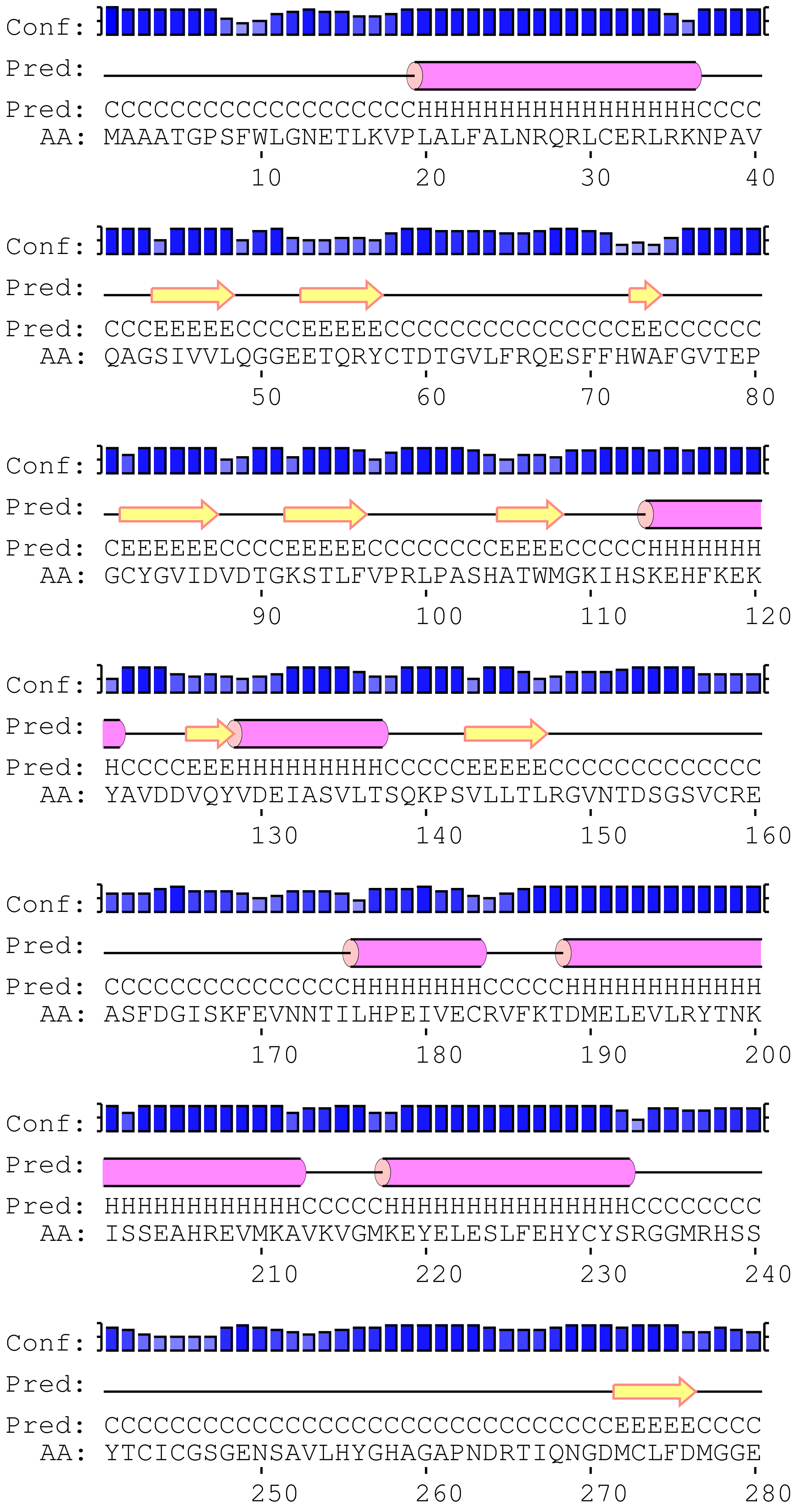
Conf:
Pred:
Pred: CCCCCCCCCCCCCCCCCCCHHHHHHHHHHHHHHHHHCCCC
AA: MAAATGPSFWLGNETLKVPLALFALNRQRLCERLRKNPAV
10 20 30 40
Conf:
Pred:
Pred: CCCEEEEECCCCEEEEECCCCCCCCCCCCCCCEECCCCCC
AA: QAGSIVVLQGGEETQRYCTDTGVLFRQESFFHWAFGVTEP
50 60 70 80
Conf:
Pred:
Pred: CEEEEEECCCCEEEEECCCCCCCCEEEECCCCCHHHHHHH
AA: GCYGVIDVDTGKSTLFVPRLPASHATWMGKIHSKEHFKEK
90 100 110 120
Conf:
Pred:
Pred: HCCCCEEEHHHHHHHHHCCCCEEEEECCCCCCCCCCCCCC
AA: YAVDDVQYVDEIASVLTSQKPSVLLTLRGVNTDSGSVCRE
130 140 150 160
Conf:
Pred:
Pred: CCCCCCCCCCCCCCCHHHHHHHHCCCCCHHHHHHHHHHHH
AA: ASFDGISKFEVNNTILHPEIVECRVFKTDMELEVLRYTNK
170 180 190 200
Conf:
Pred:
Pred: HHHHHHHHHHHHCCCCCHHHHHHHHHHHHHHHCCCCCCCC
AA: ISSEAHREVMKAVKVGMKEYELESLFEHYCYSRGGMRHSS
210 220 230 240
Conf:
Pred:
Pred: CCCCCCCCCCCCCCCCCCCCCCCCCCCCCCCEEEEECCCC
AA: YTCICGSGENSAVLHYGHAGAPNDRTIQNGDMCLFDMGGE
250 260 270 280

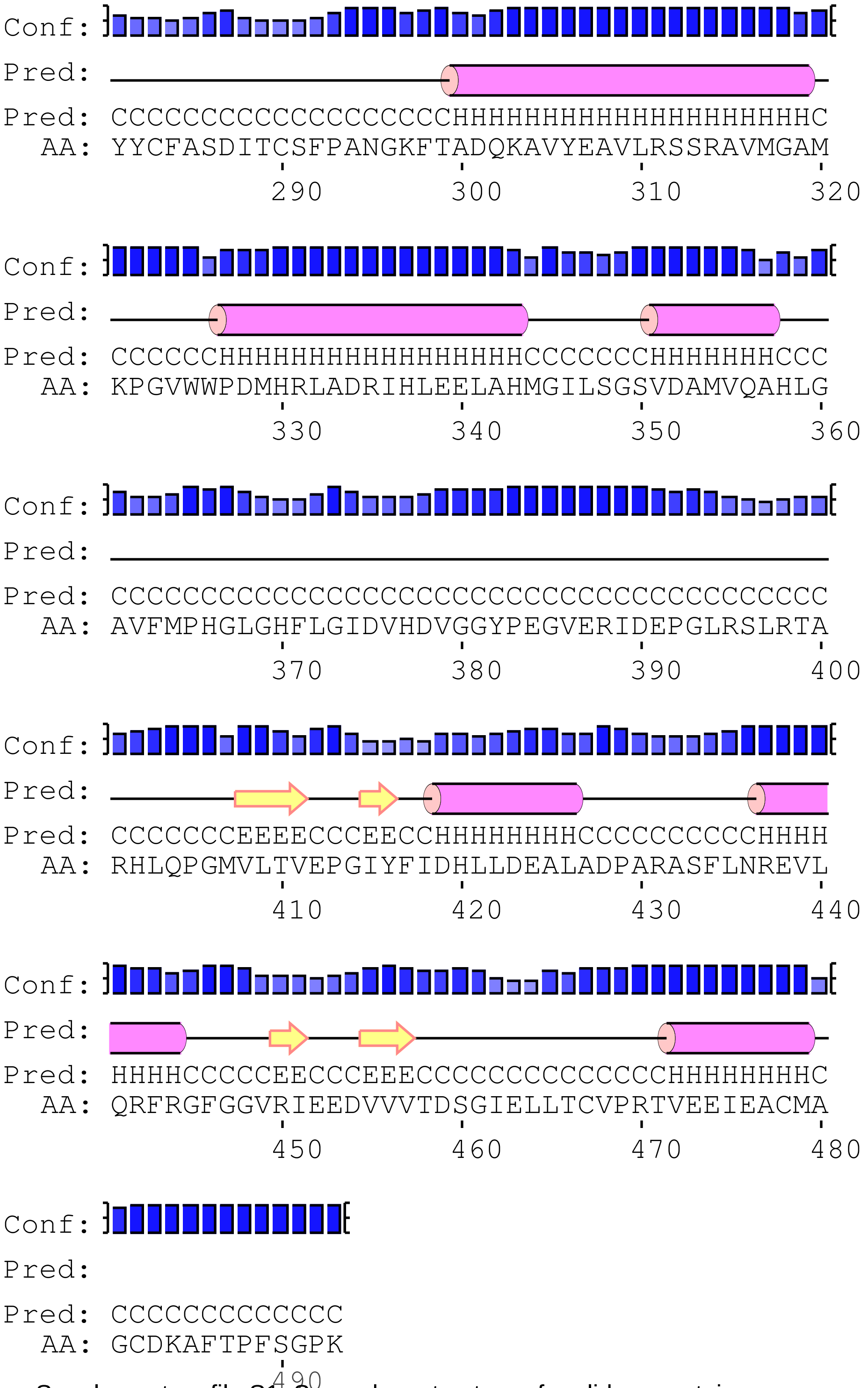

Supplementary file S1: Secondary structure of prolidase protein

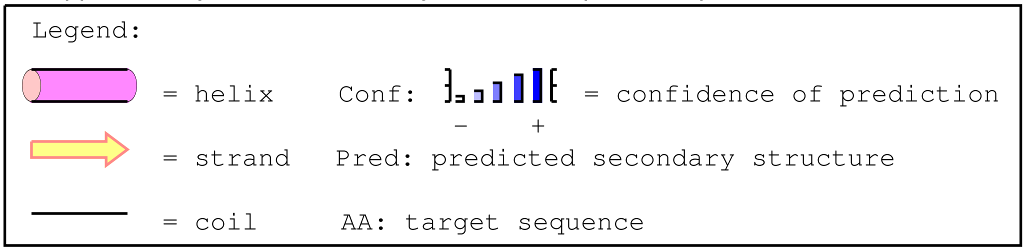

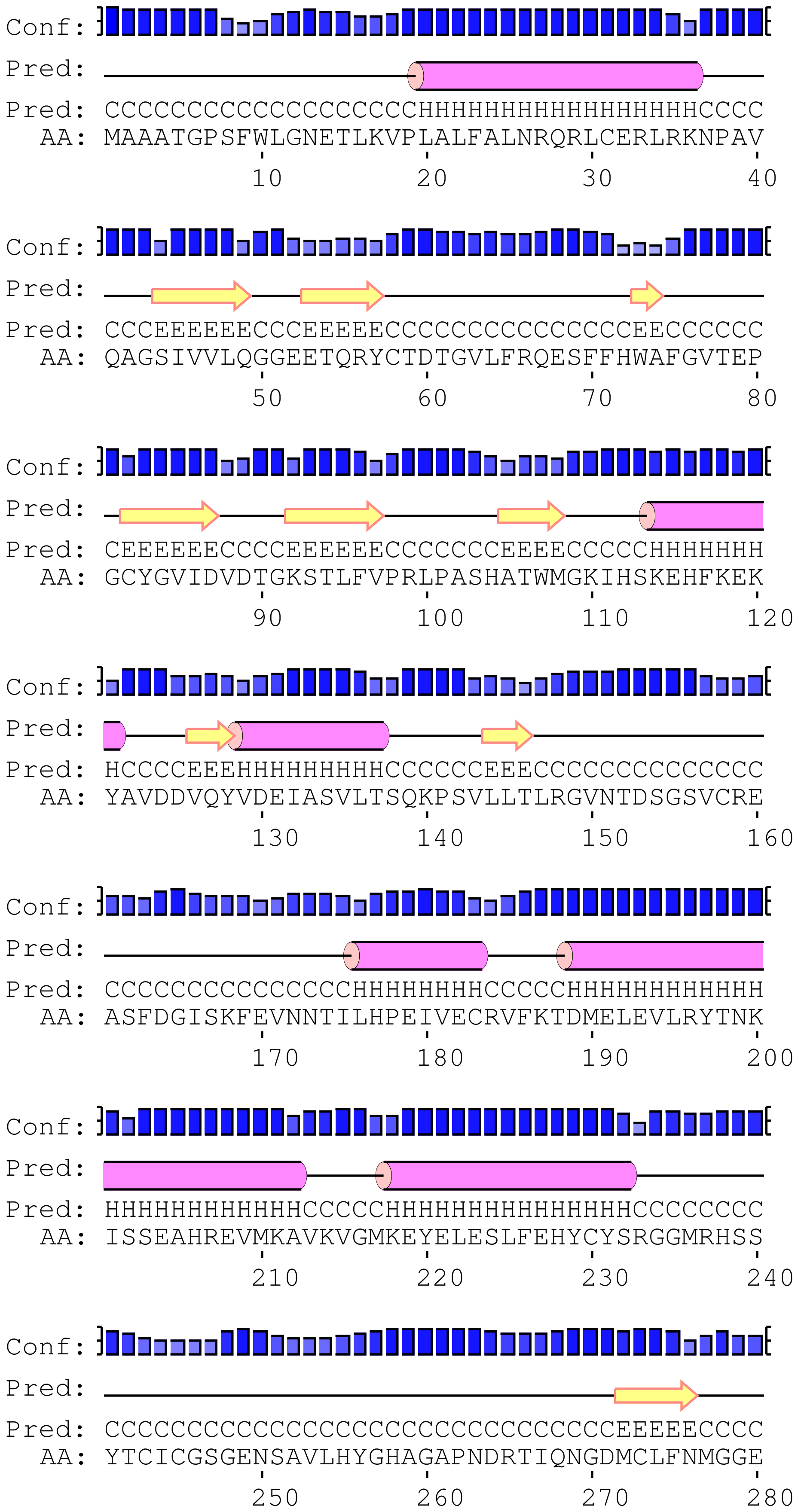
Conf:
Pred:
Pred: CCCCCCCCCCCCCCCCCCCHHHHHHHHHHHHHHHHHCCCC
AA: MAAATGPSFWLGNETLKVPLALFALNRQRLCERLRKNPAV
10 20 30 40
Conf:
Pred:
Pred: CCCEEEEEECCCEEEEECCCCCCCCCCCCCCEECCCCCCC
AA: QAGSIVVLQGGEETQRYCTDTGVLFRQESFFHWAFGVTEP
50 60 70 80
Conf:
Pred:
Pred: CEEEEEECCCCEEEEEECCCCCCCEEEECCCCCHHHHHHH
AA: GCYGVIDVDTGKSTLFVPRLPASHATWMGKIHSKEHFKEK
90 100 110 120
Conf:
Pred:
Pred: HCCCCEEEHHHHHHHHHCCCCCCCEEECCCCCCCCCCCCC
AA: YAVDDVQYVDEIASVLTSQKPSVLLTLRGVNTDSGSVCRE
130 140 150 160
Conf:
Pred:
Pred: CCCCCCCCCCCCCCCHHHHHHHHCCCCCHHHHHHHHHHHH
AA: ASFDGISKFEVNNTILHPEIVECRVFKTDMELEVLRYTNK
170 180 190 200
Conf:
Pred:
Pred: HHHHHHHHHHHHCCCCCHHHHHHHHHHHHHHHCCCCCCCC
AA: ISSEAHREVMKAVKVGMKEYELESLFEHYCYSRGGMRHSS
210 220 230 240
Conf:
Pred:
Pred: CCCCCCCCCCCCCCCCCCCCCCCCCCCCCCCEEEEECCCC
AA: YTCICGSGENSAVLHYGHAGAPNDRTIQNGDMCLFNMGGE
250 260 270 280

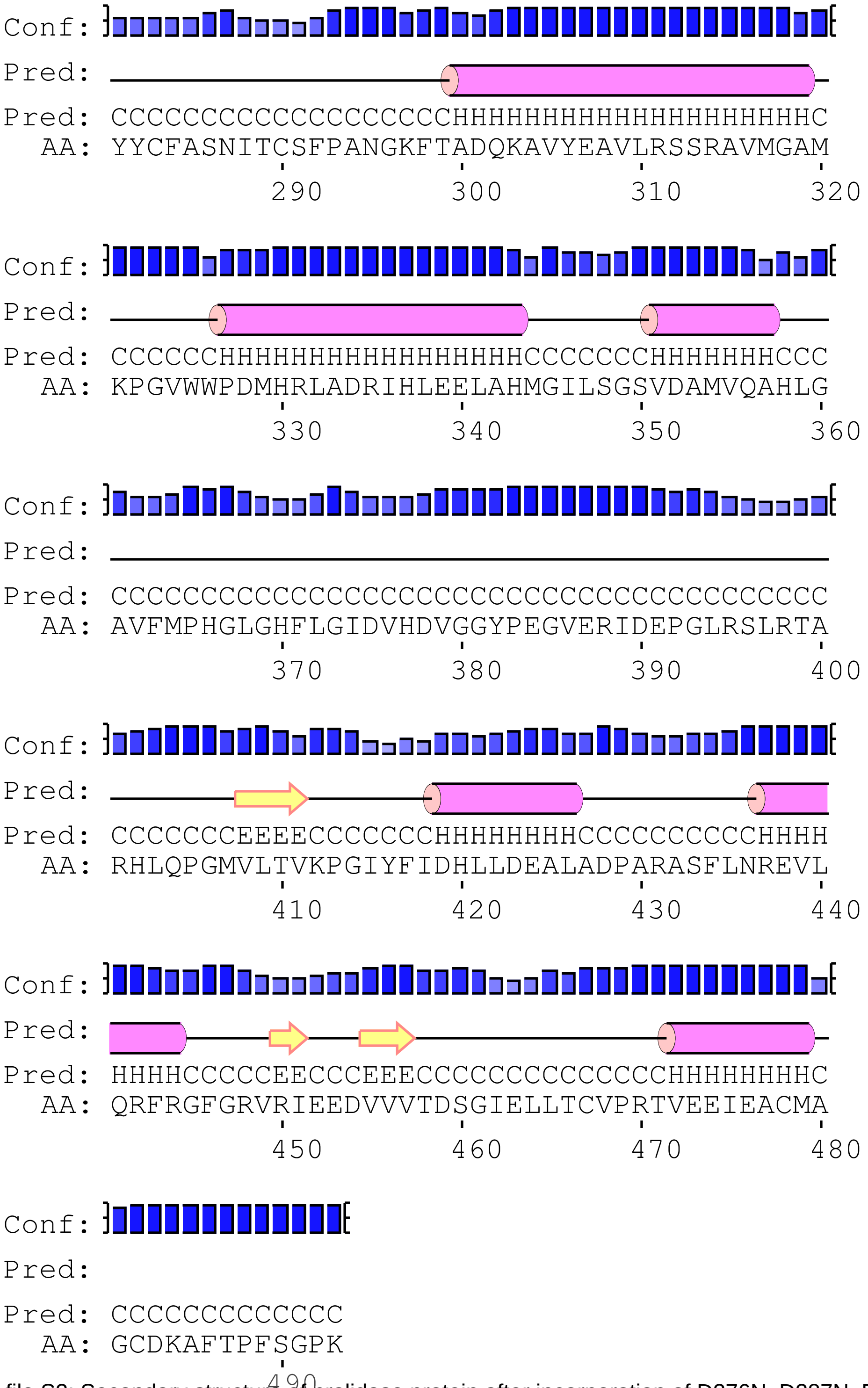

Supplementary file S2: Secondary structure of prolidase protein after incorporation of D276N, D287N, E412K, G448R substitutions

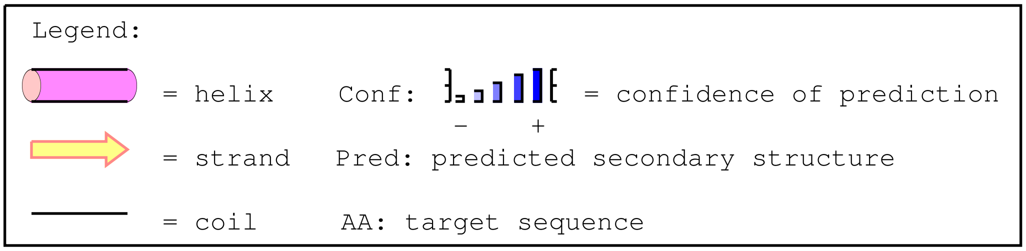

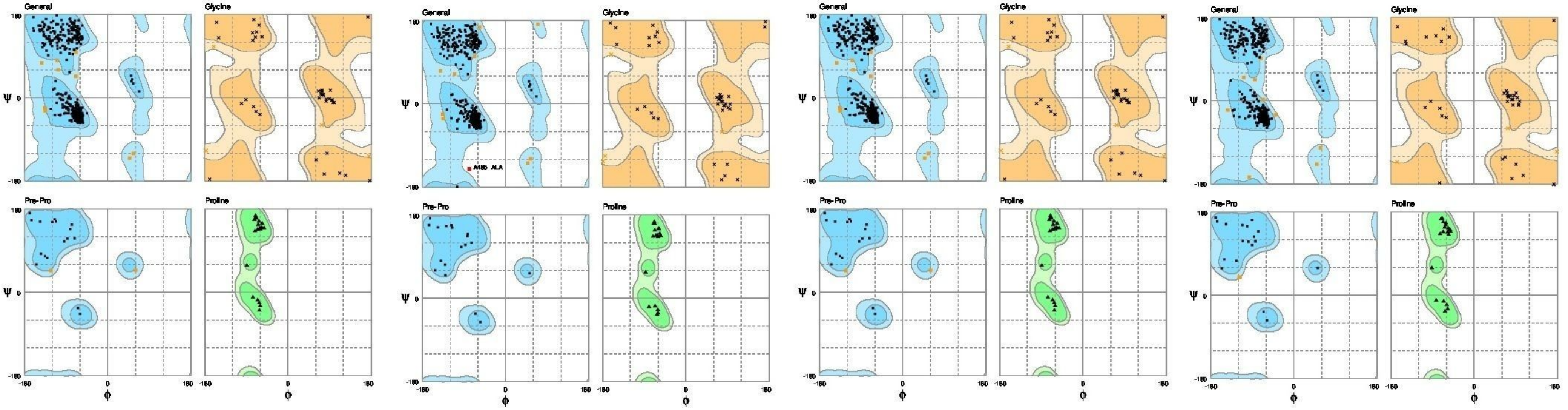

Supplementary figure File 3. a D276N RAMPAGE VALIDATION b. D287N RAMPAGE VALIDATION c. E412K RAMPAGE VALIDATION d. G448R RAMPAGE VALIDATION
